# Promoting the key moments of hand hygiene in a Targeted Hygiene intervention at COP26: Lisa Ackerley

**DOI:** 10.1093/eurpub/ckac131.345

**Published:** 2022-10-25

**Authors:** A Paskey, L Ackerley, C Buckley, S Cooper, S Bainbridge

**Affiliations:** 1 Medical Sciences - Germ Protection, Reckitt, Montvale, USA; 2 Medical and Scientific Engagement, Reckitt, Slough, UK; 3 Medical Sciences - Germ Protection, Reckitt, Hull, UK; 4 New Growth Platforms, Reckitt, Slough, UK

## Abstract

The COP26 conference in Glasgow, November 2021 presented a unique public health challenge in the midst of the COVID-19 pandemic. With 38,457 delegates attending, this international event posed a risk for SARS-CoV-2 infection. We used Targeted Hygiene theory to carry out a large event risk assessment process that focused on how spaces were used, referred to evidence from scientific literature, and identified key moments for surface and hand hygiene interventions. We relied on behavioural science evidence to optimize hand hygiene compliance at the event. To do so, we secured the opportunity to use hand sanitizer by determining the most suitable locations for over 500 hand sanitizing stations. To further motivate uptake of personal hygiene, kits were provided for each delegate with hygiene messaging to improve knowledge of the importance of hand hygiene in breaking the chain of infection. A COP26-branded face covering, personal hand gel and wipes were provided in the pack. Training and cleaning protocols centred on Targeted Hygiene were developed for cleaning teams to implement. Compliance monitoring through observation and daily real-time reporting of over 250 adenosine triphosphate (ATP) checks on just-cleaned surfaces provided reassurance for public health agencies that our Targeted Hygiene approach was effective.

The messaging and cleaning interventions were carried out for the duration of the COP26 conference. The number of individuals officially affiliated with COP26 that tested positive was ∼2 in 1,000 as compared to ∼11-12 in 1,000 individuals in Scotland during the same period (6-13 November 2021 as reported by Public Health Scotland). Whilst no single control can be attributed to this achievement, effective hand and surface hygiene interventions contributed by helping to break the chain of infection. This risk-based approach to Targeted Hygiene serves as a blueprint for effective, sustainable and measurable nonpharmaceutical interventions at large scale events.

**Key messages:**

• To mitigate risk of infection at COP26, key moments for surface and hand hygiene were identified and emphasized in cleaning protocols and education.

• Using a risk-based approach to Targeted Hygiene serves as a blueprint for effective, sustainable and measurable nonpharmaceutical interventions at large scale events such as COP26.

